# High Mobility Group Box 1 (HMGB1) Induces Toll-Like Receptor 4-Mediated Production of the Immunosuppressive Protein Galectin-9 in Human Cancer Cells

**DOI:** 10.3389/fimmu.2021.675731

**Published:** 2021-06-21

**Authors:** Anette Teo Hansen Selnø, Stephanie Schlichtner, Inna M. Yasinska, Svetlana S. Sakhnevych, Walter Fiedler, Jasmin Wellbrock, Steffen M. Berger, Elena Klenova, Bernhard F. Gibbs, Elizaveta Fasler-Kan, Vadim V. Sumbayev

**Affiliations:** ^1^ Medway School of Pharmacy, Universities of Kent and Greenwich, Chatham Maritime, United Kingdom; ^2^ Department of Oncology, Hematology and Bone Marrow Transplantation With Section Pneumology, Hubertus Wald University Cancer Center, University Medical Center Hamburg-Eppendorf, Hamburg, Germany; ^3^ Department of Pediatric Surgery, Department of Biomedical Research, Children's Hospital, Inselspital, University of Bern, Bern, Switzerland; ^4^ Department of Biomedical Research, University of Bern, Bern, Switzerland; ^5^ School of Biological Sciences, University of Essex, Colchester, United Kingdom; ^6^ Division of Experimental Allergy and Immunodermatology, University of Oldenburg, Oldenburg, Germany; ^7^ Department of Biomedicine, University of Basel and University Hospital Basel, Basel, Switzerland

**Keywords:** galectin-9, TGF-beta 1, HMGB1 (high mobility group box 1), immune surveillance, Toll-like receptors

## Abstract

High mobility group box 1 (HMGB1) is a non-histone protein which is predominantly localised in the cell nucleus. However, stressed, dying, injured or dead cells can release this protein into the extracellular matrix passively. In addition, HMGB1 release was observed in cancer and immune cells where this process can be triggered by various endogenous as well as exogenous stimuli. Importantly, released HMGB1 acts as a so-called “danger signal” and could impact on the ability of cancer cells to escape host immune surveillance. However, the molecular mechanisms underlying the functional role of HMGB1 in determining the capability of human cancer cells to evade immune attack remain unclear. Here we report that the involvement of HMGB1 in anti-cancer immune evasion is determined by Toll-like receptor (TLR) 4, which recognises HMGB1 as a ligand. We found that HGMB1 induces TLR4-mediated production of transforming growth factor beta type 1 (TGF-β), displaying autocrine/paracrine activities. TGF-β induces production of the immunosuppressive protein galectin-9 in cancer cells. In TLR4-positive cancer cells, HMGB1 triggers the formation of an autocrine loop which induces galectin-9 expression. In malignant cells lacking TLR4, the same effect could be triggered by HMGB1 indirectly through TLR4-expressing myeloid cells present in the tumour microenvironment (e. g. tumour-associated macrophages).

## Introduction

High mobility group box 1 (HMGB1) is a non-histone protein predominantly localised in the cell nucleus where it promotes nuclear transcription processes by interacting with DNA (1–4). However, HMGB1 can be passively released into the extracellular matrix by dead, dying or injured cells (1–4). It can also be secreted by cancer and immune cells in response to various exogenous or endogenous stimuli (1–4). Secreted HMGB1 acts as a “danger signal” or “alarmin” which may also trigger malignant tumour progression (1–4). We have recently reported that HMGB1 promotes the generation and secretion of interleukin-1beta (IL-1β), which induces production of stem cell factor (SCF) by competent cells (5, 6). As such, it could facilitate progression of malignancies such as acute myeloid leukaemia (AML) (6–8).

HMGB1 was reported to act as a ligand for various immune receptors including toll-like receptors (TLRs) 2 and 4, which normally recognise pathogen-associated molecular patterns shared by Gram-negative and Gram-positive bacteria respectively (6). HMGB1 receptors also include receptor of advanced glycation end products (RAGE) and the immune receptor Tim-3 (T cell immunoglobulin and mucin domain containing protein 3) (4, 6). Through these receptors HMGB1 in its immunogenic form could trigger both pro-inflammatory and pro-angiogenic responses (6). Our recent work also suggested that, in Toll-like receptor 4 (TLR4)-expressing cells, HMGB1 induces the production and secretion of transforming growth factor beta type 1 (TGF-β) (9). TGF-β displays both autocrine and paracrine activities and, through the transcription factor Smad3, induces expression of galectin-9, an immunosuppressive protein which impairs the anti-cancer activities of natural killer and cytotoxic T cells (9, 10). Galectin-9 is known as a so-called “tandem protein” which contains two ligand/sugar-binding domains fused together by a linker peptide. As a result of alternative splicing, galectin-9 may be present in three main isoforms which differ because of the length of their linker peptide: long (49 amino acids), medium (27 amino acids) and short (15 amino acids) isoforms (11–13).

Currently, the molecular mechanisms underlying the role of the immunogenic form of HMGB1 in determining the capability of human cancer cells to escape host immune surveillance remain unclear. We hypothesised that HMGB1 could upregulate galectin-9 expression in cancer cells through the activation of TGF-β production induced in a TLR4-dependent manner. Here we report for the first time that the role of HMGB1 in anti-cancer immune evasion is determined by the TLR4. Cancer cells expressing TLR4 release TGF-β in response to stimulation with HMGB1 followed by TGF-β-induced upregulation of galectin-9 expression, forming such an HMBG1-triggered autocrine loop. Galectin-9 expression in cancer cells lacking functional TLR4 could still be triggered by HMGB1 indirectly *via* induction of TGF-β expression in TLR4-positive myeloid cells (e.g. tumour-associated macrophages) present in the tumour microenvironment. Importantly, other HMGB1 receptors did not demonstrate any involvement in the process of induction of TGF-β expression by this danger signal and its follow-up events.

## Materials and Methods

### Materials

RPMI-1640 medium for culturing the cells, foetal bovine serum, supplements and basic laboratory chemicals were purchased from Sigma (Suffolk, UK). Microtiter plates for ELISA were obtained from Oxley Hughes Ltd (London, UK). Rabbit antibodies against galectin-9, RAGE and phospho-S423/S425-Smad3 as well as mouse antibodies against TLRs 2 and 4 were purchased from Abcam (Cambridge, UK). Antibodies against β-actin were purchased from Abcam (Cambridge, UK) and Proteintech (Manchester, UK). Goat anti-mouse and anti-rabbit fluorescently-labelled dye secondary antibodies were obtained from Li-COR (Lincoln, Nebraska USA). ELISA-based assay kits for the detection of galectin-9, Tim-3 and TGF-β as well as human recombinant TGF-β1 protein were purchased from Bio-Techne (R&D Systems, Abingdon, UK). ELISA kits for the detection of HMGB1 were purchased from MyBioSource (San Diego, CA. USA). Human HMGB1 and anti-Tim-3 mouse monoclonal antibody were described before (6, 14). All other chemicals purchased were of the highest grade of purity commercially available.

### Cell Lines and Primary Human Samples

Cell lines used in this work were purchased from either the European Collection of Cell Cultures (THP-1, Colo-205 and MCF-7) or the American Tissue Culture Collection (ATTC – HEK293). Cell lines were accompanied by identification test certificates and were grown according to corresponding tissue culture collection protocols.

For description of primary cells (9, 15–17) please see the Ethics statement.

### Plasmids

Plasmid encoding constitutively active human TLR4 (murine CD4 fused to human TLR4) (18) was generously provided by Professor Medzhitov (Yale University, New Haven, USA).

### Cell Transfection

mCD-hTLR4 expression plasmid or empty expression vector were transfected into Colo 205 cells using DOTAP transfection reagent according to the manufacturer’s protocol.

### Western Blot Analysis

Galectin-9, Tim-3, phospho-S423/S425 Smad-3, RAGE, TLRs 2 and 4 were measured by Western blot and compared to the amounts of β-actin (protein loading control), as previously described (18).

Li-Cor goat secondary antibodies conjugated with infrared fluorescent dyes, were used as described in the manufacturer’s protocol in order to visualise specific proteins (Li-Cor Odyssey imaging system was employed). Western blot data were quantitatively analysed using Odyssey software and values were subsequently normalised against those of β-actin or total protein loaded.

### Enzyme-Linked Immunosorbent Assays (ELISAs)

Secreted HMGB1, TGF-β, galectin-9 and Tim-3 were measured, either in cell culture medium or in blood plasma (galectin-9 was also measured in some of the cell lysates), by ELISA using MyBioSource or R&D Systems kits (see *Materials* section) according to manufacturer’s protocols.

### Design of Nanocomplexes For Immunoprecipitation Containing Anti-HMGB1 and Anti-TGF-β Antibodies

Nanoconjugates (nanocomplexes) were designed using 30 nm gold nanoparticles (AuNPs). The synthesis of 30 nm AuNPs was performed essentially as described previously (16, 19). Briefly, 5 ml of aqueous gold (III) chloride trihydrate (10 mM) and 2.5 ml of aqueous sodium citrate (100 mM) were added to 95 ml of MilliQ-water in a round bottom flask supplied with a magnetic stirrer, and the resulting pale-yellow solution was allowed to cool to 1–2°C. Under vigorous stirring, 1 ml of chilled (4°C) aqueous 0.1 M sodium borohydride was added and the dark red solution obtained was then stirred for further 10 min in an ice bath before being allowed to warm to room temperature. The obtained AuNPs were characterised using transmission electron microscopy (TEM) as previously described (16, 20). TEM image of the AuNP is presented in the **Supplementary Figure 1**. Antibody molecules were attached as described before in the ratio of 1 AuNP: 6 antibody molecules in both cases. A sample scheme of the nanoconjugate and its action are presented in **Supplementary Figure 2**. Briefly, the antibody (biotinylated anti-HMGB1 or anti-TGF-β) was coupled to 30 nm AuNPs using glutathione (GSH, tripeptide containing γ-glutamate, cysteine-SH and glycine) as a linker. A 0.05 M solution of GSH was incubated for 2 h at room temperature with a 1 : 1 mixture of aqueous solutions of 0.4 M 1-ethyl-3-(3-dimethylpropyl)-carbodiimide (EDC) and 0.1 M N-hydroxysuccinimide (NHS) to allow the activation of GSH –COOH groups, thus enabling them to interact with antibody amino groups. Activated GSH was then mixed with an antibody in an equimolar ratio and incubated for at least 2 h at 37°C. The antibody was then mixed with 30 nm AuNPs (0.5 mM) at a ratio of 1 AuNP: 6 antibody molecules. This mixture was incubated for at least 18 h at room temperature.

The available amount of AuNPs was calculated using the following equations:

$$N_{AuNP}=m_{totalAu}/m_{AuNP}(m=mass;\;N=quantity);$$

$$m_{total}Au=C_{Au}(mol\times l^{-1})\times MWAu(g\times mol^{-1})\times V_{AuNP}$$

$$(C=molar\;concentration,\;MW=molecular\;weight,V_{AuNP}=the\;volume\;of\;the\;AuNP\;suspensionused);$$

$$m_{1\;AuNP}=\rho_{Au}\times V_{1\;AuNP};(\rho =density,\;V=volume;\rho Au=19.3\;g\;cm^{-3});$$

$$V_{1AuNP}=4/3\;\pi R^{3};(R=radius\;of\;a\;AuNP)$$

$$R=d_{1AuNP}/2;(d=diameter\;of1\;AuNP\;\text{expressed }in\;cm)$$

The amount of antibody molecules was calculated from the molar weight of the antibodies (150,000 g × mol^−1^) and the Avogadro constant so that 1 mole of an antibody contained 6.02 × 10^23^ antibody molecules.

In order to confirm successful conjugation of antibodies to the nanoparticles we used the following approaches.

Anti-HMGB1 antibody-conjugated and naked (control) AuNPs were exposed to HRP-conjugated streptavidin for 30 min followed by precipitation by centrifugation (16). Nanomaterials were washed three times with bi-distilled water and exposed for 2 min exposure to 6 mg/ml o-Phenylenediamine (OPD) in the presence of hydrogen peroxide. The reaction was stopped using 10% sulfuric acid. Nanomaterials were precipitated by centrifugation (16) and the absorbance of supernatants was analysed at 492 nm (**Supplementary Figure 3**).

Mouse anti-human TGF-β1-conjugated AuNPs were exposed for 1 h to anti-mouse or anti-rabbit (control) fluorescent dye-labelled Li-Cor secondary antibodies. nanomaterials were then precipitated by centrifugation (16), washed three times with bi-distilled water and analysed using Li-Cor Odyssey C_LX_ imager (**Supplementary Figure 4**).

### Statistical Analysis

Each experiment was performed at least three times and statistical analysis, when comparing two normally distributed events at a time, was conducted using a two-tailed Student’s *t*-test. In cases when multiple comparisons (more than two groups) were performed, we used an ANOVA test. Post-hoc Bonferroni correction was used where applicable. Statistical probabilities (p) were expressed as * when p<0.05; **, p<0.01 and *** when p<0.001.

## Results

**Figure 1:**
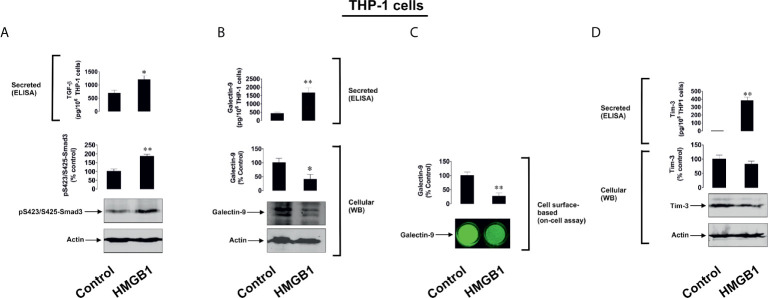
HMGB1 induces TGF-β dependent expression of galectin-9 in THP-1 human AML cells. THP1 cells were exposed for 24 h to 1 µg/ml HMGB1 followed by measurement of TGF-β secretion and Smad3 activating phosphorylation **(A),** galectin-9 protein expression and secretion **(B)**, galectin-9 cell surface presence **(C)** and Tim-3 protein expression and release **(D)**. Images are from one experiment representative of 4 giving similar results. Quantitative data are shown as mean values ± SEM from 4 independent experiments. *p < 0.05 and **p < 0.01 *vs* control.

**Figure 2:**
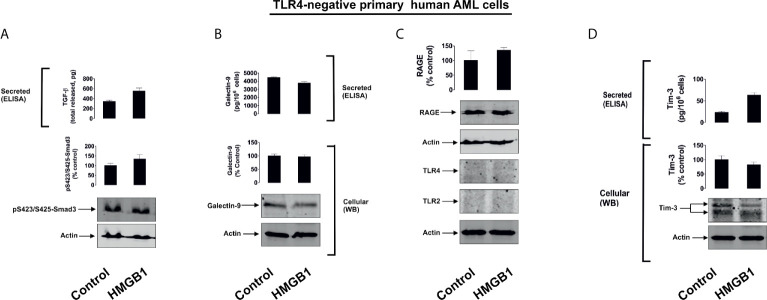
HMGB1 does not induce TGF-β secretion and galectin-9 expression in primary human AML cells lacking expressions of TLRs 2 and 4. Primary human AML cells lacking expression of TLRs 2 and 4 were exposed for 16 h to 2.5 µg/ml HMGB1 and TGF-β secretion and Smad3 activating phosphorylation **(A)**, galectin-9 expression and secretion **(B)**, expression of TLRs 2 and 4 as well as RAGE proteins **(C)** and Tim-3 protein expression and secretion **(D)** were analysed. Images are from one experiment representative of 4 which gave similar results. Quantitative data are shown as mean values ± SEM from four independent experiments.

Firstly, we investigated human monocytic AML THP-1 cells and exposed them for 24 h to 1 µg/ml of immunogenic form of human recombinant HMGB1 (6). We found that exposure to HMGB1 upregulated TGF-β secretion, which subsequently triggered Smad3 phosphorylation (**Figure 1A**). This led to a substantial increase in galectin-9 secretion and reduced levels of total cell-associated and cell surface-based galectin-9 (**Figures 1B, C**). Overall, an increase in total amounts of galectin-9 produced by THP-1 cells was observed and also confirmed by measuring the total quantities of galecting-9 present in cell lysates and secreted protein in cell culture medium by ELISA. Resting cells produced 890 ± 48 pg/10^6^ cells galectin-9 in total and this rose to 2110 ± 93 pg/10^6^ cells in HMGB1-treated cells. The reduction in cell surface galectin-9 expression suggests its proteolytic shedding off the cell surface together with Tim-3 (which acts both as a receptor and possible trafficker for galectin-9) (21). An increase in Tim-3 secretion was also observed (**Figure 1D**), which is known to be governed by proteolytic shedding triggered by TLR4 signalling (22). THP-1 cells express four HMGB1 receptors (6) – TLRs 2 and 4, receptor of advanced glycation end products (RAGE) and Tim-3 (**Supplementary Figure 5**). To confirm the role of the TLRs 2 and 4 in observed responses we studied primary human AML cells obtained from newly diagnosed patients. We selected patients where AML cells expressed Tim-3 and RAGE, but did not express (as determined by Western blotting) detectable amounts of TLRs 2 and 4 proteins, and exposed these AML cells to 2.5 µg/ml HMGB1 for 16 h. None of the effects observed in THP-1 cells were detected in these primary AML cells (**Figure 2**). These results suggest that TLRs and not RAGE or Tim-3 are mediating HMGB1-induced TGF-β secretion and follow-up events.

Interestingly, primary human AML cells expressing TLR4 responded to 16 h exposure to 2.5 µg/ml HMGB1 in a similar manner to that observed for THP-1 cells – where the secretion of both galectin-9 and Tim-3 was significantly upregulated (**Supplementary Figure 6**).

**Figure 3:**
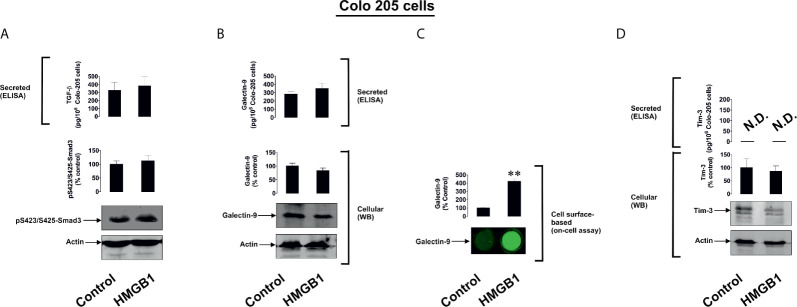
The effects of HMGB1 on Galectin-9 and Tim-3 expression in Colo 205 human colorectal cancer cells. Colo 205 cells were exposed for 24 h to 1 µg/ml HMGB1 followed by measurement of TGF-β secretion and Smad3 activating phosphorylation **(A)**, galectin-9 protein expression and secretion **(B)**, galectin-9 cell surface presence **(C)** and Tim-3 protein expression and release **(D)**. Images are from one experiment representative of four which gave similar results. Quantitative data are expressed as mean values ± SEM of four independent experiments. **p < 0.01 *vs* control. N.D. in this and other figures denotes “not detectable”.

**Figure 4:**
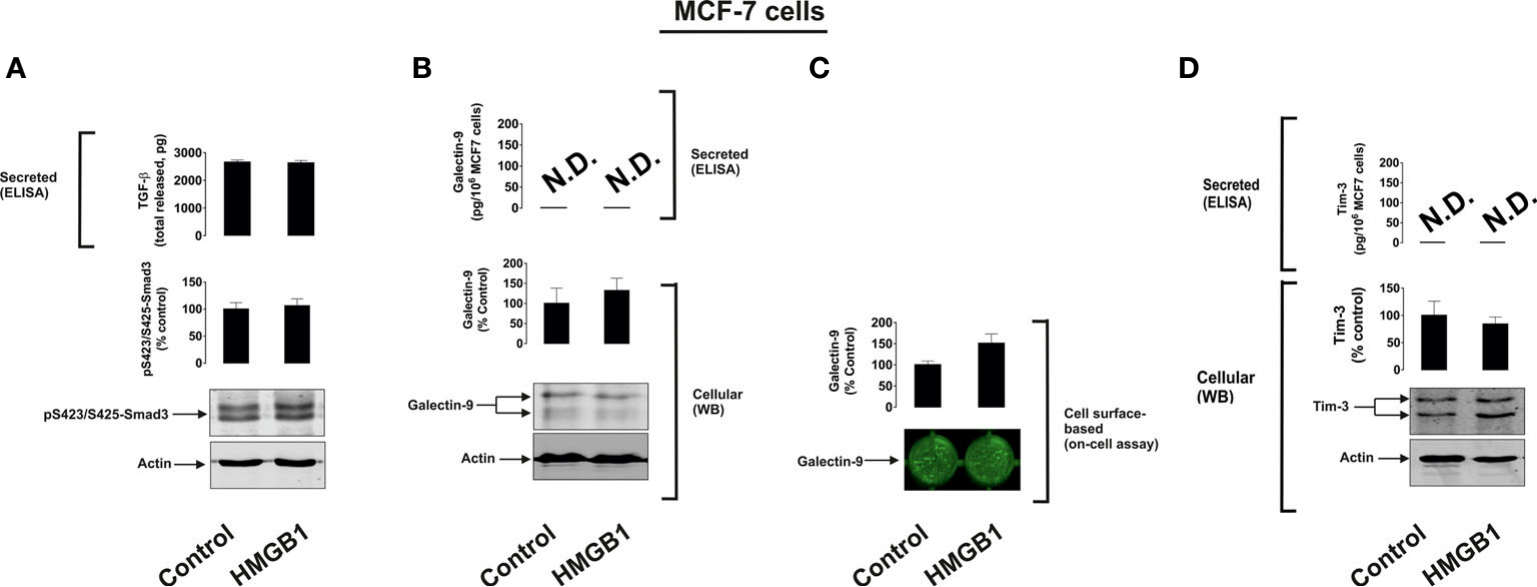
The effects of HMGB1 on Galectin-9 and Tim-3 expression in MCF-7 human breast cancer cells. MCF-7 cells were exposed for 24 h to 1 µg/ml HMGB1 followed by measurement of TGF-β secretion and Smad3 activating phosphorylation **(A)**, galectin-9 protein expression and secretion **(B)**, galectin-9 cell surface presence **(C)** and Tim-3 protein expression and release **(D)**. Images are from one experiment representative of four which gave similar results. Quantitative data are shown as mean values ± SEM of four independent experiments.

**Figure 5:**
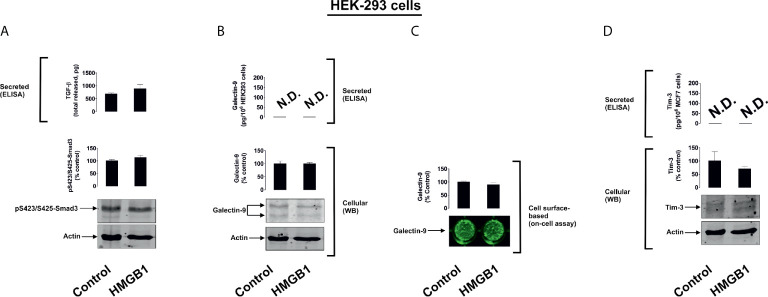
The effects of HMGB1 on Galectin-9 and Tim-3 expression in HEK293 human embryonic kidney cells. MCF-7 cells were exposed for 24 h to 1 µg/ml HMGB1 followed by measurement of TGF-β secretion and Smad3 activating phosphorylation **(A)**, galectin-9 protein expression and secretion **(B)**, galectin-9 cell surface presence **(C)** and Tim-3 protein expression and release **(D)**. Images are from one experiment representative of four which gave similar results. Quantitative data are shown as mean values ± SEM of four independent experiments.

To further investigate the differential roles of TLRs 2 and 4 in the observed effects of HMGB1, we studied human colorectal cancer cells (Colo 205), human breast cancer cells (MCF-7) and human embryonic kidney cells (HEK293). Colo 205 expressed TLR2, RAGE and Tim-3, whereas the other cell types investigated expressed RAGE and Tim-3, but not the other HMGB1 receptors – TLR4 and TLR2 (**Supplementary Figure 5**). Of all the different cell types investigated, upregulation of galectin-9 translocation onto the cell surface was only observed in Colo 205 cells (these cells express TLR2) upon exposure to 1 µg/ml HMGB1 for 24 h, but neither total galectin-9 levels nor its secretion were upregulated in these cells (**Figure 3**). Other effects which were observed in THP-1 cells, e.g. activation of TGF-β production, Smad-3 phosphorylation and upregulated Tim-3 secretion, were undetectable in Colo 205 cells (**Figure 3**). In other cell types – MCF-7 (**Figure 4**) and HEK293 (**Figure 5**) HMGB1 did not trigger any changes observed in THP-1 cells. Taken together, these results suggest that TLR4 mediates HMGB1-induced TGF-β production followed by its autocrine and paracrine effects.

**Figure 6:**
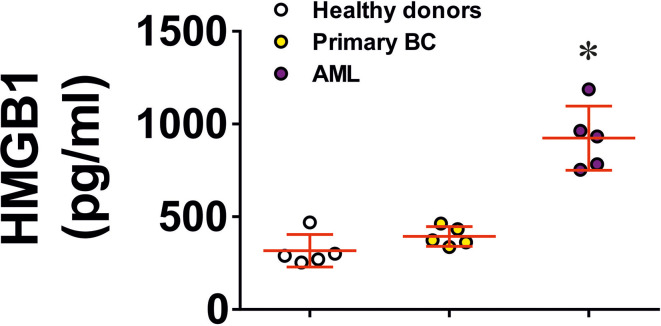
HMGB1 levels in blood plasma of human healthy donors and cancer patients. HMGB1 levels were detected in blood plasma of 5 healthy donors, 5 primary breast cancer (BC) patients, 5 metastatic breast cancer patients and 5 AML patients. Data are mean values ± SEM of 5 independent experiments. Individual values for each patient are shown as well. *p < 0.05 *vs* healthy donors.

We then tested if naturally occurring, dying cell-derived HMGB1 can induce similar effects in TLR4 expressing cells. We exposed several types of human cancer cell lines (THP-1, Colo 205 and MCF-7) for 24 h to 100 µM BH3I-1 (5-[(4-bromophenyl)methylene]-a-(1-methylethyl)-4-oxo-2-thioxo-3-thiazolidineacetic acid), which is a synthetic cell-permeable Bcl-XL antagonist that induces apoptosis (by inhibiting the interactions between the BH3 domain and Bcl-XL thus defunctionalising mitochondria) (23). We found that upon stimulation with BH3I-1 THP-1 released the highest amounts of HMGB1 (**Supplementary Figure 7**). To confirm the physiological relevance of this observation, we compared HMGB1 levels in the blood plasma of healthy donors, primary breast cancer patients (a solid tumour where HMGB1 release was assessed in breast cancer-derived MCF-7 cell lines) and AML patients (a “liquid” tumour which was also tested using the THP-1 cell line). We tested the blood plasma of 5 healthy donors, 5 AML patients and 5 patients with primary breast cancer. Here, we observed that AML patients had significantly higher levels of HMGB1 in their blood plasma compared to healthy donors and primary breast cancer patients (**Figure 6**). These results suggest that in the studied solid tumours HMGB1, if released, most likely stays within the tumour microenvironment.

**Figure 7:**
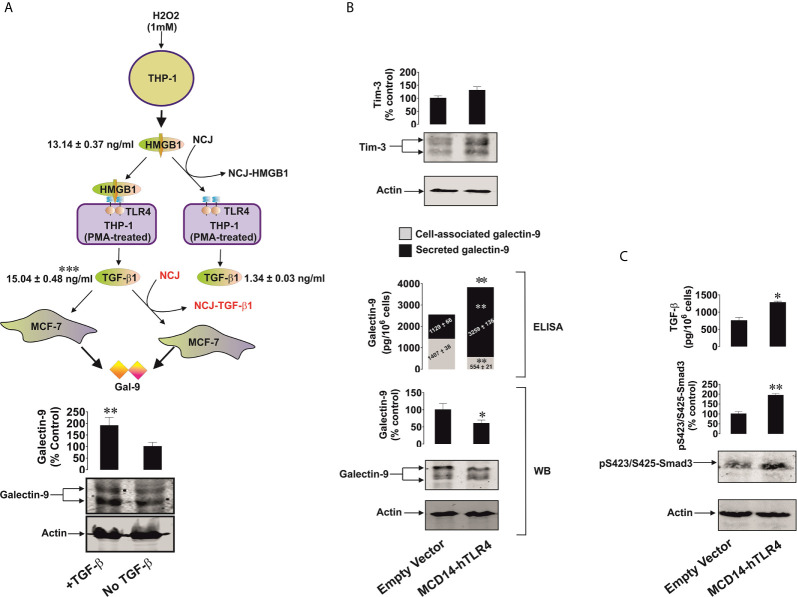
TGF-β produced by TLR4 expressing cells upon stimulation with HMGB1 triggers galectin-9 expression in target cancer cells. Active TLR4 is capable of inducing the same effects without HMGB1. **(A)** THP-1 cells were treated with 1 mM H_2_O_2_ for 24 h and the medium was collected for further use. This medium after H_2_O_2_ degradation with or without immunoprecipitation of HMGB1 was used to treat PMA-differentiated THP-1 cells expressing high levels of TLR4 for 24 h. The medium from PMA-treated THP-1 cells treated with HMGB1-containing medium was collected. This medium with or without immunoprecipitation of TGF-β was used to incubate MCF-7 cells for 24 h followed by measurement of galectin-9 by Western blot. **(B)** Colo 205 cells were transfected with constitutively active TLR4 or corresponding empty vector followed by 24h of incubation without further treatment. Expression of Tim-3 protein and it’s secretion (values are presented in the text), galectin-9 protein expression by both Western blot and ELISA and secretion by ELISA were analysed. **(C)** TGF-β secretion and Smad3 activating phosphorylation were also detected. Images are from one experiment representative of 4 giving similar results. Quantitative data are shown as mean values ± SEM from 4 independent experiments. *p < 0.05 and **p < 0.01 *vs* control.

Given the results described above, THP-1 monocytic cells were chosen to act as HMGB1 releasers and were exposed to 1 mM hydrogen peroxide for 24 h and released 13.14 ± 0.37 ng/ml HMGB1 (**Figure 7A**). We used this medium (H_2_O_2_ was degraded using 5 mg/ml horseradish peroxidase) to treat THP-1 cells, which were pre-treated for 24 h with 100 nM phorbol 12-myristate 13-acetate (PMA), in order to differentiate them into macrophages which express high levels of TLR4 (24). As a control, we exposed PMA-pre-treated THP-1 cells to the same medium but depleted from HMGB1 by immunoprecipitating HMGB1 using nanoconjugates (NCJ, 30 nm gold nanoparticles carrying antibodies against HMGB1) as outlined in Materials in Methods. We found that HMGB1-containing medium led to the production of *ca.* 15 ng/ml TGF-β. This result suggests that naturally produced cell-derived HMGB1 displays higher biological activity than the recombinant protein. The effect was not observed in the cells treated with medium where HMGB1 was removed (**Figure 7A**). TGF-β-containing medium was then used to treat MCF-7 human breast cancer cells. As a control, we exposed MCF-7 cells to the same medium where TGF-β was immunoprecipitated using NCJ (30 nm gold nanoparticles carrying antibody against TGF-β) as described in Materials and Methods. We found that TGF-β-containing medium significantly upregulated galectin-9 levels in MCF-7 cells (**Figure 7A**).

We then sought to further confirm the ability of the TLR4 to mediate the observed effects. For this purpose we transfected Colo 205 cells, where we could not detect TLR4 protein by Western blot (**Supplementary Figure 5**), with constitutively active TLR4 (mCD4-hTLR4, the construct which consists of the human transmembrane and intracellular domains of TLR4 fused to the extracellular domain of mouse CD4, kindly provided by Prof Ruslan Medzhitov, Yale University, USA) (18). The presence of mCD4-hTLR4 resulted in the upregulation of galectin-9 secretion and reduced its cell-associated levels. We additionally measured cell-associated galectin-9 by ELISA and observed that it was significantly downregulated in the cells transfected with mCD4-hTLR4, while total galectin-9 levels (secreted + cell-associated) were significantly upregulated in mCD4-hTLR4-transfected cells (**Figure 7B**). Tim-3 secretion of 95 ± 9 pg/10^6^ cells was also observed in the presence of mCD4-hTLR4 (**Figure 7B**) while in the cells which did not contain mCD4-hTLR4 it was undetectable. Furthermore, mCD4-hTLR4 upregulated TGF-β secretion, which led to the upregulation of activating Smad3 phosphorylation in these cells (**Figure 7C**).

## Discussion

**Figure 8:**
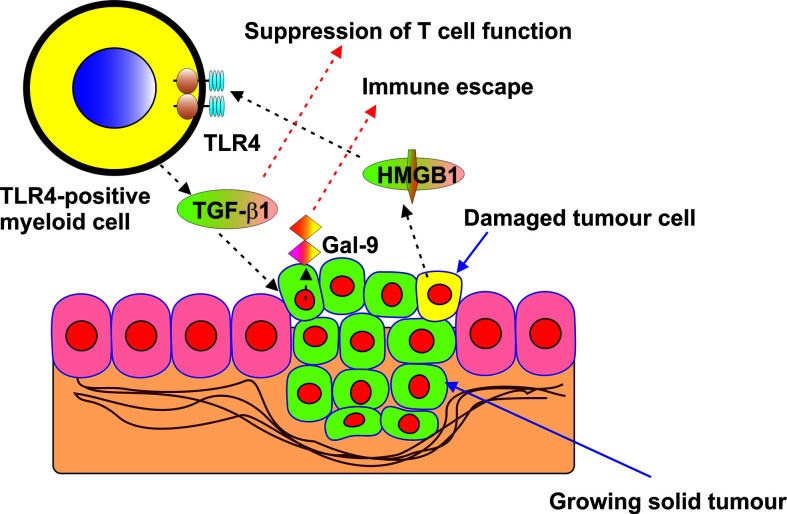
Proposed mechanism of indirect upregulation of galectin-9 expression by HMGB1 in solid tumour cells lacking TLR4 expression is presented in the scheme.

The role of HMGB1 as a “danger signal” or alarmin in cancer progression has been extensively studied in the last decade (1–4, 6–8). It has become increasingly evident that it could play a role in anti-cancer immune evasion but the biochemical mechanisms underlying this phenomenon remain unclear. In this work we discovered that in cancer cells expressing TLR4, HMGB1 can induce TGF-β production and secretion (**Figure 1**). Interestingly, TLR4 has recently been shown to mediate TGF-β production when activated by lipopolysaccharide (LPS), its pathogen-associated ligand, and some endogenous stimuli such as hyaluronic acid (25, 26). It was also demonstrated that HMGB1-induced activation of TGF-β production in TLR4-expressing cells takes place through the activator protein 1 (AP-1) transcription factor and its upstream pathway (9). HMGB1 displays TLR4 ligand properties and could act in TLR4-expressing cells similarly to that observed for LPS (6, 9). An additional factor which could mediate HMGB1-induced TGF-β expression in TLR4 expressing cells could be the hypoxia-inducible factor 1 (HIF-1) transcription complex, which is upregulated by TLR4-mediated downstream signalling (24). HIF-1 was shown to trigger the expression of TGF-β (9). TGF-β secreted by TLR4-expressing cancer cells (in our case THP-1 human AML cells), displays autocrine/paracrine activities, and thus acts on the malignant cells which have produced it. As a result, TGF-β induces galectin-9 production by these cells in a Smad3-dependent manner, the effect which has recently been reported (9) and confirmed in the present study. Galectin-9 subsequently displays immunosuppressive activities on cytotoxic and helper T cells in the tumour microenvironment as well as on NK cells (10, 17). The effects of HMGB1-induced galectin-9 expression were observed in the TLR4-expressing THP-1 human AML cell line (**Figure 1**) as well as in primary human AML cells expressing this receptor (**Supplementary Figure 6**). Our further experiments demonstrated that if cells do not express TLR4, but express other HMGB1 receptors (TLR2, Tim-3 and RAGE), no induction of TGF-β secretion takes place in these cells when exposed to HMGB1. This was the case in primary human AML cells lacking TLR4 expression (**Figure 2**), Colo 205 (**Figure 3**), MCF-7 (**Figure 4**) and HEK293 cells (**Figure 5**) and, as a result, no subsequent upregulation of galectin-9 expression was observed in these cells. However, HMGB1 can still upregulate TGF-β production in TLR4-expressing cells (e. g. tumour-associated macrophages) present in the tumour microenvironment where the released TGF-β may activate galectin-9 expression in cancer cells. Furthermore, increased TGF-β in the tumour microenvironment contributes to the creation of an immunosuppressive milieu (27). TGF-β suppresses granzyme B expressions in cytotoxic T cells, thus reducing their cell-killing potential and impairing their anti-cancer activity (28). Furthermore, TGF-β is known to contribute to the differentiation of naïve T cells entering the tumour microenvironment into regulatory T cells (Tregs) which suppress the cytotoxic activities of T cells (29). This proposed pathway is demonstrated using the example of a solid tumour in **Figure 8**.

Taken together, our results suggest that HMGB1 potentially plays a critical role in anti-cancer immune evasion and these effects are facilitated by TLR4.

## Data Availability Statement

The original contributions presented in the study are included in the article/**Supplementary Material**. The datasets used and/or analyzed during the current study are available from the corresponding author on reasonable request.

## Ethics Statement

Blood plasma of healthy human donors was obtained, from buffy coat blood (from healthy donors undergoing routine blood donation) (15) which was purchased from the National Health Blood and Transfusion Service (NHSBT, UK) following ethical approval (REC reference: 16-SS-033). Primary human AML plasma samples and cells [cultured as described before (16)] were obtained from the sample bank of University Medical Centre Hamburg-Eppendorf (Ethik Kommission der Ärztekammer Hamburg, reference: PV3469). Primary human AML mononuclear blasts (AML-PB001F, newly diagnosed/untreated) were also purchased from AllCells (Alameda, CA, USA) and handled in accordance with the manufacturer’s recommendations. The studies were performed following ethical approval (REC reference: 16-SS-033). Blood samples were collected before breast surgery from patients with primary breast cancer (PBC) and before treatment of patients who had metastatic breast cancer (MBC). Samples were also collected from healthy donors (individuals with no diagnosed pathology – see above). Blood separation was performed using a buoyancy density method employing Histopaque 1119-1 (Sigma, St. Louis, MO) according to the manufacturer’s protocol (9, 17). Ethical approval for these studies was obtained from the NRES Essex Research Ethics Committee and the Research & Innovation Department of the Colchester Hospitals University, NHS Foundation Trust [MH 363 (AM03) and 09/H0301/37]. Written informed consent for participation was not required for this study in accordance with the national legislation and the institutional requirements.

## Author Contributions

AT, SS, IY and SSS performed all the reported experiments with equal contributions. WF and JW isolated and provided primary AML samples used to obtain crucial data and shared expertise on handling primary human AML cells. EK provided primary blood samples from breast cancer patients and shared expertise on their handling. SB participated in design of the concept and planning the experiments. BG, EF-K, and VS designed the study, planned all the experiments, analyzed the data, and wrote the manuscript. All authors contributed to the article and approved the submitted version.

## Funding

This work was supported in part by the Batzebär grant (to EF-K and SB). The funders had no role in study design, data collection, data analysis, interpretation, or writing of the report.

## Conflict of Interest

The authors declare that the research was conducted in the absence of any commercial or financial relationships that could be construed as a potential conflict of interest.
