# Supplementary material for: High Mobility Group Box 1 (HMGB1) Induces Toll-Like Receptor 4-Mediated Production of the Immunosuppressive Protein Galectin-9 in Human Cancer Cells
Source: Front Immunol. 2021 Jun 21;12:675731. doi: 10.3389/fimmu.2021.675731 (PMC8255966; doi:10.3389/fimmu.2021.675731)
Supplement: Supplementary file 1 [file DataSheet_1.docx]

**Supplementary Figures**

**High mobility group box 1 (HMGB1) induces Toll-like receptor 4-mediated production of the immunosuppressive protein galectin-9 in human cancer cells**

Anette Teo Hansen Selnø, Stephanie Schlichtner, Inna M. Yasinska, Svetlana S. Sakhnevych, Walter Fiedler, Jasmin Wellbrock, Steffen M. Berger, Elena Klenova, Bernhard F. Gibbs, Elizaveta Fasler-Kan and Vadim V. Sumbayev


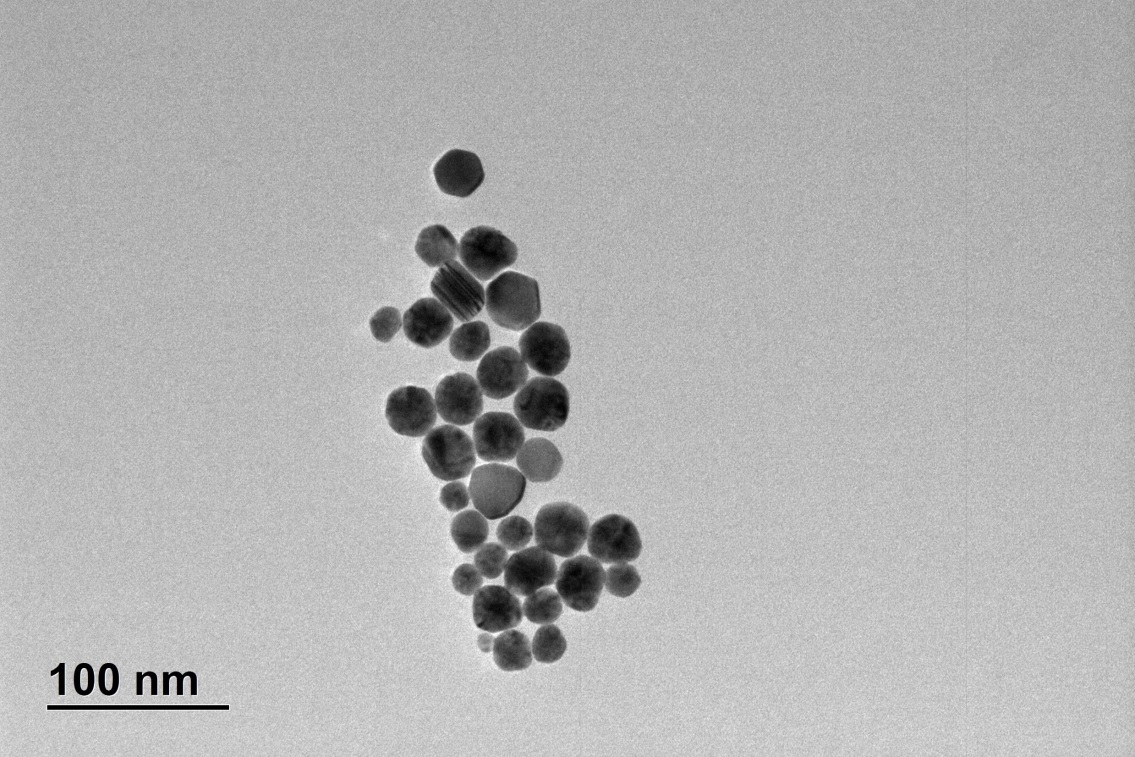


**Supplementary figure 1. TEM image of AuNPs used to design nanoconjugates for HMGB1 immunoprecipitation.** The average size of the nanoparticles was also measured by TEM.

**
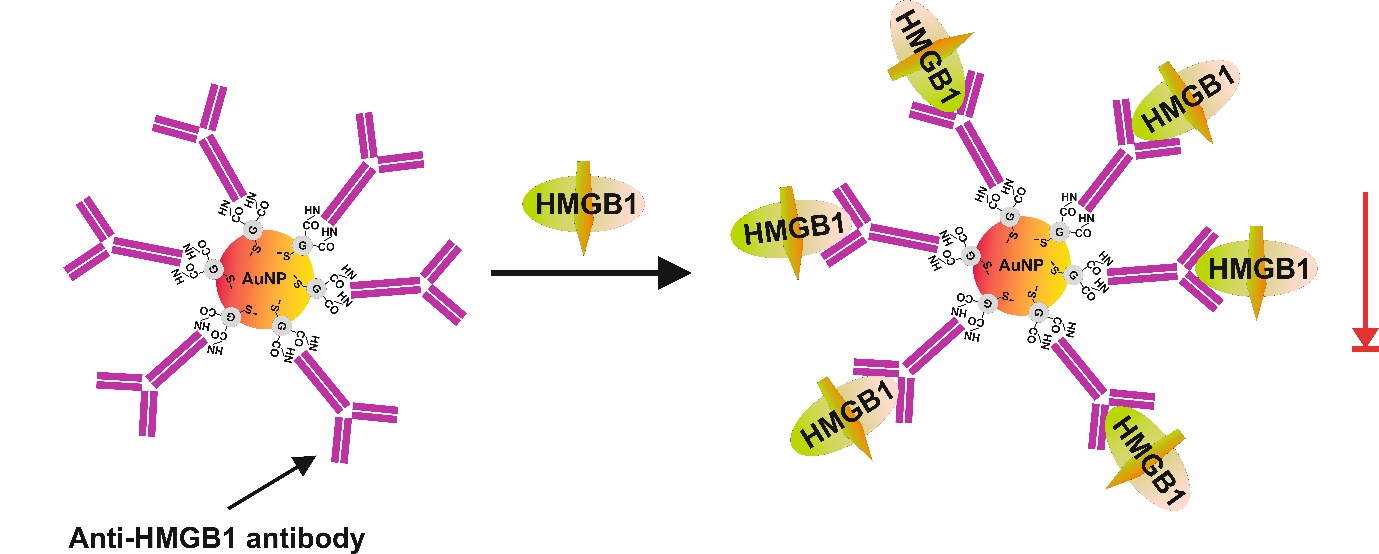
**

**Supplementary figure 2. Scheme of the nanoconjugate designed to immunoprecipitate HMGB1 from cell culture medium.** Design is described in Materials and Methods.


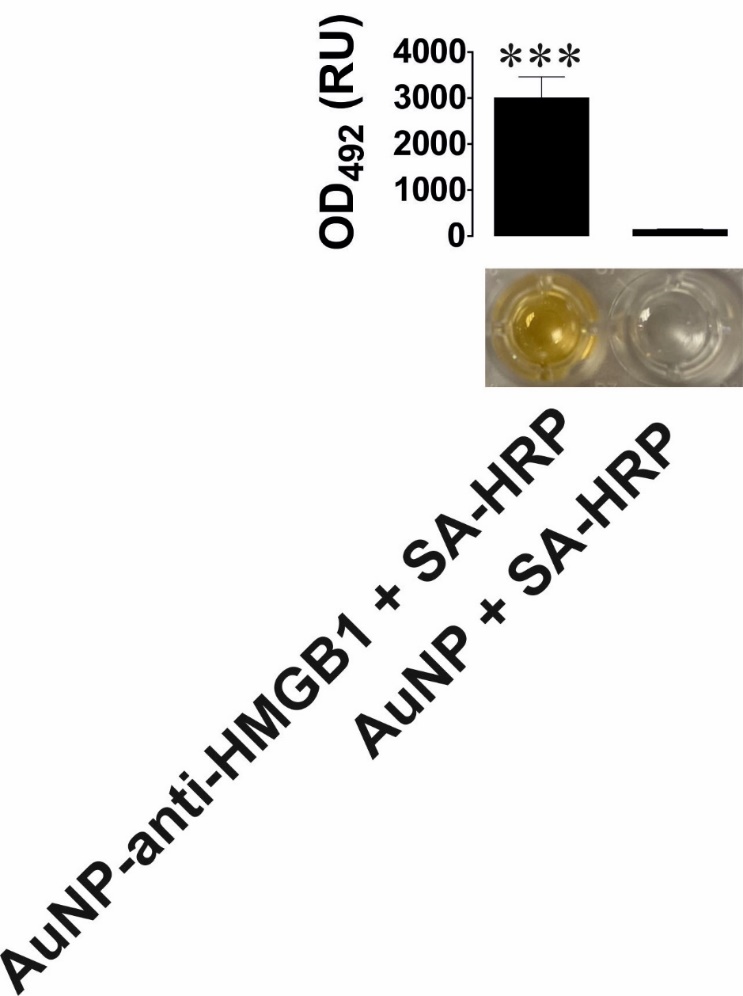


**Supplementary figure 3. Biotinylated anti-HMGB1 antibody is attached to the gold nanoparticles (AuNPs).** Anti-HMGB1 antibody conjugated and naked (control) AuNPs were exposed to HRP-conjugated streptavidin for 30 min followed by precipitation and analysis. The depicted image is from one experiment representative of three which gave similar results. Quantitative data are shown as mean values ± SEM of three independent experiments.

**
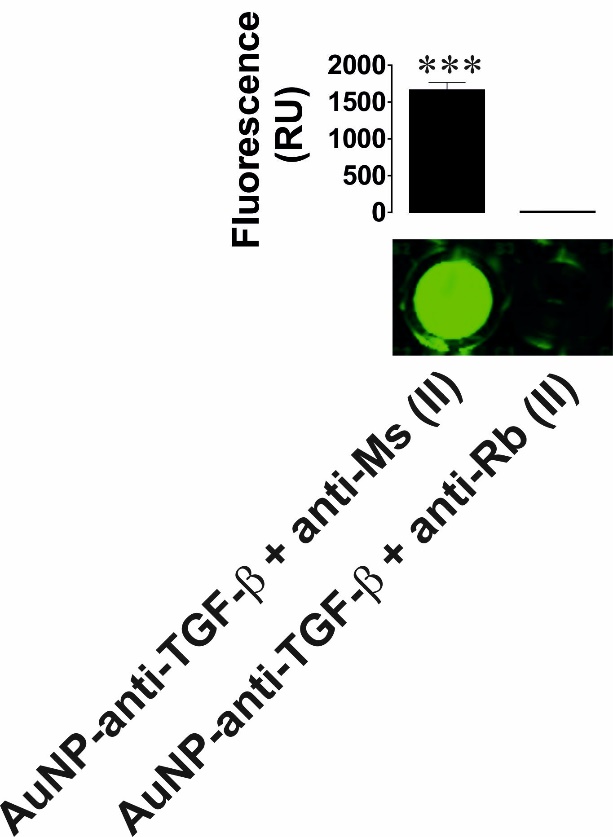
**

**Supplementary figure 4. Mouse anti-human TGF-β1 antibody is attached to the gold nanoparticles (AuNPs).** Anti-TGF-β antibody conjugated AuNPs were exposed for 1 h to anti-mouse and anti-rabbit (control) fluorescently labelled Li-Cor secondary antibodies, followed by precipitation and analysis using an Odyssey C_LX_ imager. The image shown is from one experiment representative of three which gave similar results. Quantitative data are shown as mean values ± SEM of three independent experiments.

**A**

**
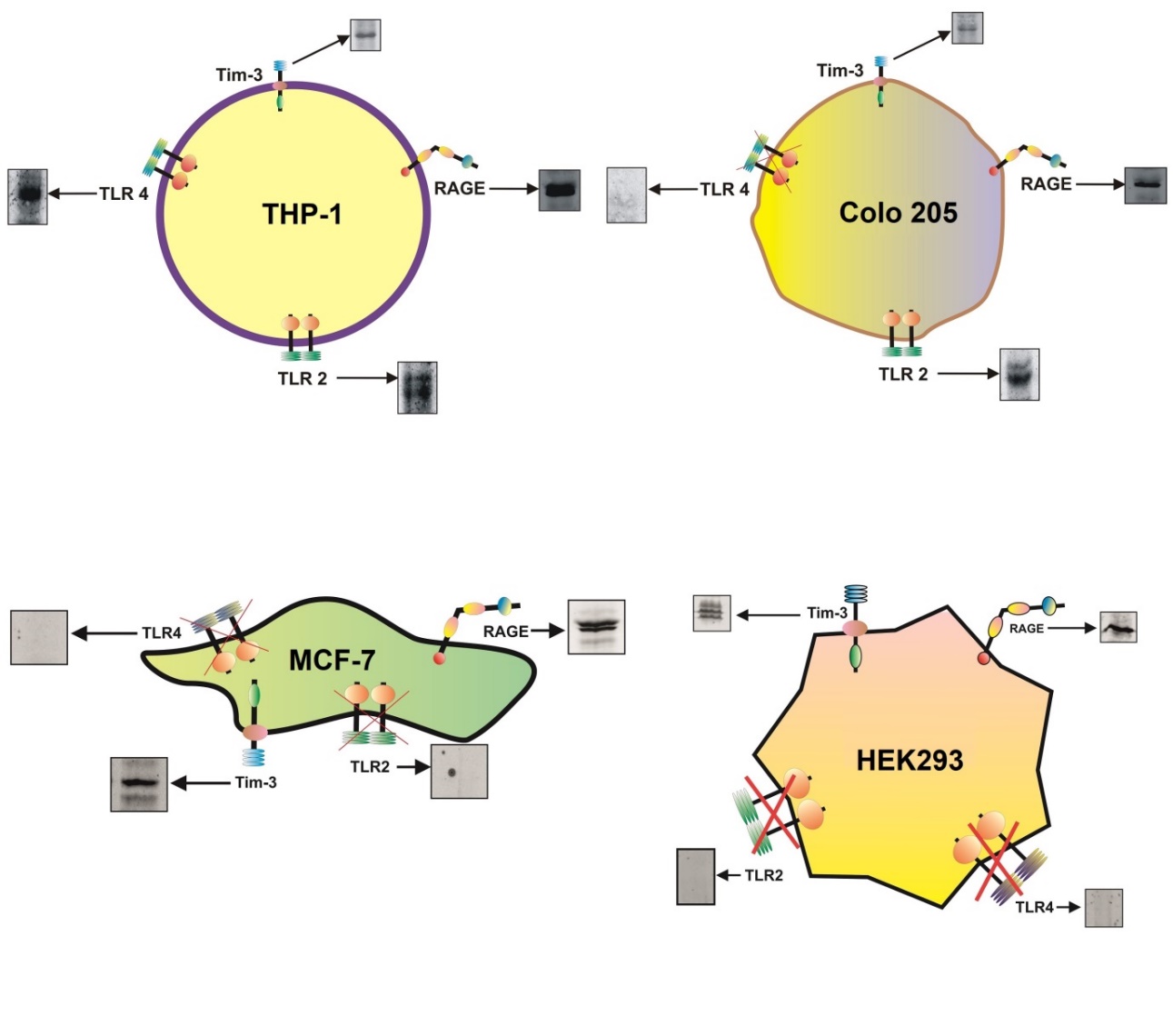
**

**B**

| **HMGB1 receptor**  **Cell line** | **TLR4** | **TLR2** | **Tim-3** | **RAGE** |
| --- | --- | --- | --- | --- |
| **THP-1 (AML)** | **+** | **+** | **+** | **+** |
| **Colo 205 (Colorectal cancer)** | **-** | **+** | **+** | **+** |
| **MCF-7 (Breast cancer)** | **-** | **-** | **+** | **+** |
| **HEK293 (Embryo)** | **-** | **-** | **+** | **+** |

**Supplementary figure 5. Expression of HMGB1 receptors – TLR4, TLR2, RAGE and Tim-3 in various human cell lines employed in the present study. (A)** Expression of indicated proteins was detected in the cell lines using Western blot analysis as outlined in the Materials and Methods (50 µg per well protein were loaded in each case). **(B)** Table summarising presence of HMGB1 receptors in the studied cell lines. Images are from one experiment representative of 4 which gave similar results.


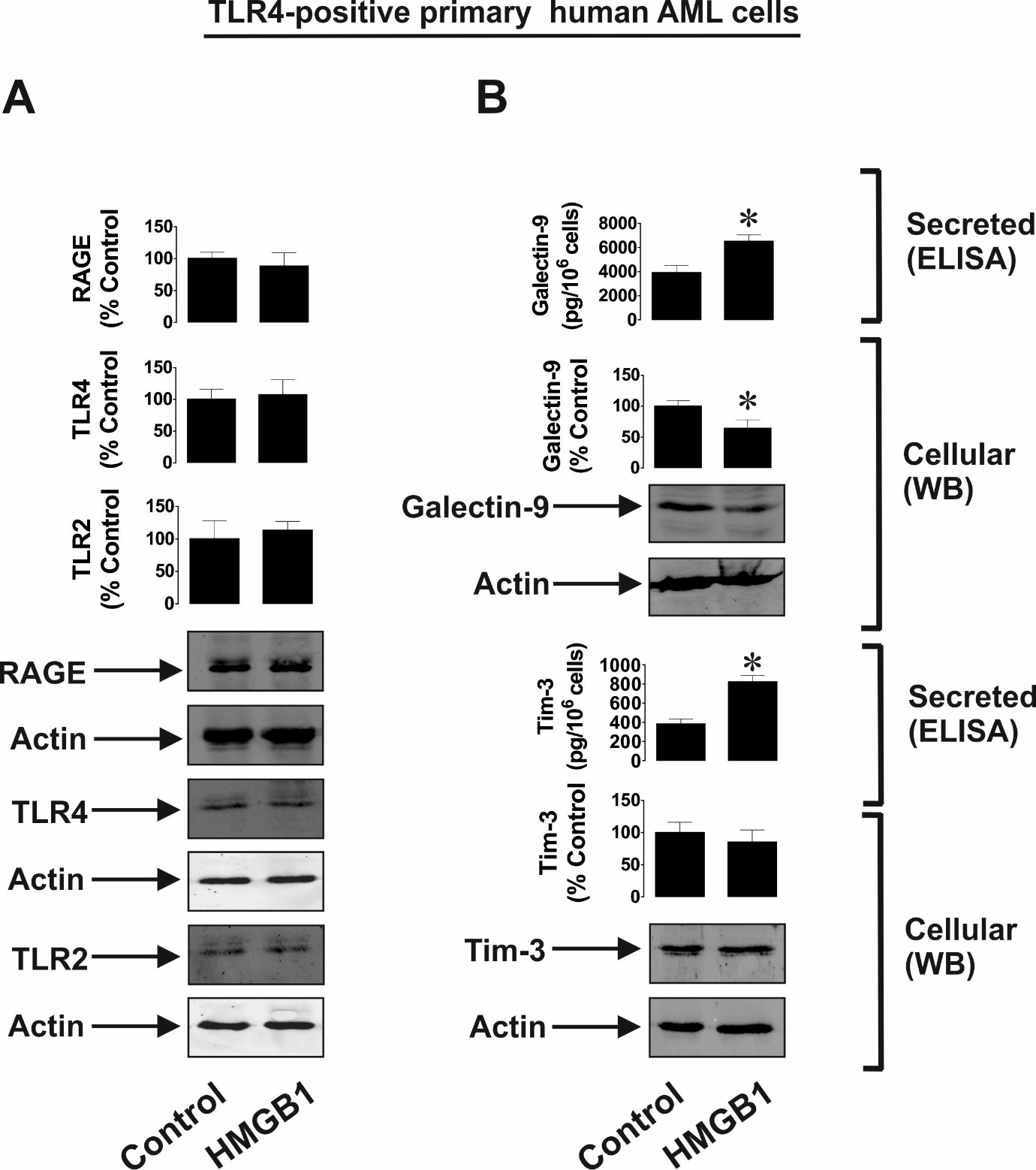


**Supplementary figure 6. HMGB1 induces Tim-3 and galectin-9 secretion in TLR4-expressing primary human AML cells.** Primary human AML cells expressing TLRs 2 and 4, as well as Tim-3 and RAGE, were exposed for 16 h to 2.5 µg/ml HMGB1. This was followed by measurements of TLR4, TLR2 and RAGE expression by Western blot **(A)**. Expression of Tim-3 and galectin-9 were also analysed by Western blot and their secreted levels quantifiedby ELISA **(B)**. Images are from one experiment representative of 3 which gave similar results. Quantitative data are shown as mean values ± SEM from three independent experiments.

**
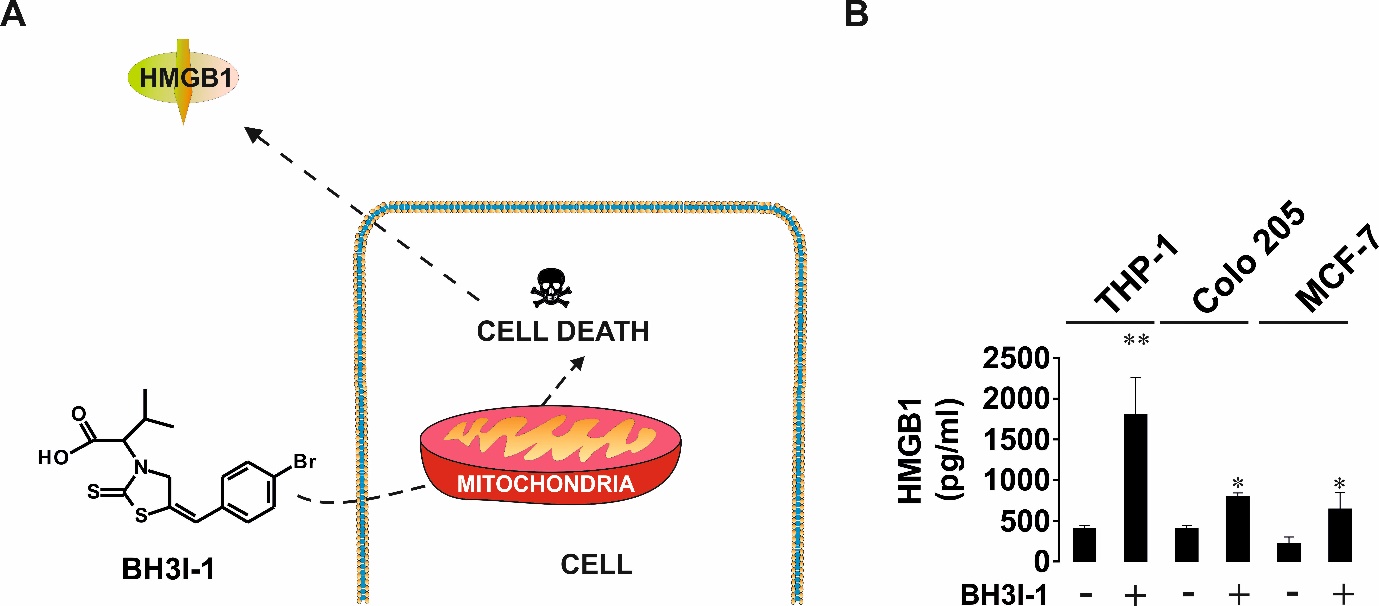
**

**Supplementary figure 7. Apoptosis inducer BH3I-1 triggers HMGB1 release from human cancer cells.** THP-1, Colo 205 and MCF-7 cells were exposed to 100 µM BH3I-1 for 24 h **(A)** followed by detection of HMGB1 release by ELISA **(B)** as outlined in the Materials and Methods. Data are mean values ± SEM of three independent experiments. * - p < 0.05 and ** - p < 0.01 *vs* control.
